# Supplementary material for: Modeling glioblastoma heterogeneity as a dynamic network of cell states
Source: Mol Syst Biol. 2021 Sep 16;17(9):e10105. doi: 10.15252/msb.202010105 (PMC8444284; doi:10.15252/msb.202010105)
Supplement: Supplementary file 6 — Source Data for Figure 5 [file MSB-17-e10105-s004.zip › Figure5A_sourcedata/GSEA_3071/nosigdb_stateIV.GseaPreranked.1622795164626/gsea_report_for_na_neg_1622795164626.html]

Report for na\_neg 1622795164626 [GSEA]

| GS  follow link to MSigDB | GS DETAILS | SIZE | ES | NES | NOM p-val | FDR q-val | FWER p-val | RANK AT MAX | LEADING EDGE || 1 | DOROSZKO\_U3013MG\_CLUSTER\_2 | Details ... | 175 | -0.67 | -3.89 | 0.000 | 0.000 | 0.000 | 1289 | tags=79%, list=24%, signal=101% |
| 2 | LARSSON\_STATE\_4 | Details ... | 87 | -0.76 | -3.89 | 0.000 | 0.000 | 0.000 | 1057 | tags=87%, list=20%, signal=107% |
| 3 | DOROSZKO\_U3180MG\_CLUSTER\_3 | Details ... | 375 | -0.59 | -3.76 | 0.000 | 0.000 | 0.000 | 1348 | tags=67%, list=25%, signal=84% |
| 4 | DOROSZKO\_U3054MG\_CLUSTER\_4 | Details ... | 456 | -0.55 | -3.62 | 0.000 | 0.000 | 0.000 | 1598 | tags=70%, list=30%, signal=92% |
| 5 | IVY\_CTMVP | Details ... | 87 | -0.68 | -3.52 | 0.000 | 0.000 | 0.000 | 493 | tags=52%, list=9%, signal=56% |
| 6 | SEURAT\_CELLCYCLE | Details ... | 87 | -0.67 | -3.42 | 0.000 | 0.000 | 0.000 | 1390 | tags=85%, list=26%, signal=113% |
| 7 | RICHARDS\_ZHONG\_NATURE\_2018\_NPCS\_UPREG | Details ... | 116 | -0.63 | -3.40 | 0.000 | 0.000 | 0.000 | 1071 | tags=74%, list=20%, signal=91% |
| 8 | FELDMAN\_2020\_G1 | Details ... | 129 | -0.62 | -3.39 | 0.000 | 0.000 | 0.000 | 1087 | tags=76%, list=21%, signal=93% |
| 9 | ANASTASSIOU\_MULTICANCER\_INVASIVENESS\_PMID\_22208948 | Details ... | 38 | -0.79 | -3.33 | 0.000 | 0.000 | 0.000 | 305 | tags=63%, list=6%, signal=67% |
| 10 | NB\_KI\_CLUSTER5 | Details ... | 75 | -0.66 | -3.26 | 0.000 | 0.000 | 0.000 | 1390 | tags=83%, list=26%, signal=111% |
| 11 | OLSEN\_MESENCHYMAL | Details ... | 67 | -0.67 | -3.23 | 0.000 | 0.000 | 0.000 | 323 | tags=48%, list=6%, signal=50% |
| 12 | NB\_KI\_CLUSTER18 | Details ... | 70 | -0.65 | -3.15 | 0.000 | 0.000 | 0.000 | 468 | tags=51%, list=9%, signal=56% |
| 13 | WANG\_MESENCHYMAL\_PMID\_28697342 | Details ... | 31 | -0.75 | -3.05 | 0.000 | 0.000 | 0.000 | 521 | tags=61%, list=10%, signal=68% |
| 14 | SUVA\_G1S\_PMID\_31327527 | Details ... | 27 | -0.79 | -3.05 | 0.000 | 0.000 | 0.000 | 728 | tags=81%, list=14%, signal=94% |
| 15 | DALMO\_EXTINCTION\_UNPUB | Details ... | 31 | -0.75 | -3.05 | 0.000 | 0.000 | 0.000 | 521 | tags=61%, list=10%, signal=68% |
| 16 | RICHARDS\_NEFTEL\_CELL\_2019\_G1\_S | Details ... | 27 | -0.79 | -3.05 | 0.000 | 0.000 | 0.000 | 728 | tags=81%, list=14%, signal=94% |
| 17 | FELDMAN\_2020\_S | Details ... | 130 | -0.56 | -3.04 | 0.000 | 0.000 | 0.000 | 1121 | tags=65%, list=21%, signal=81% |
| 18 | FELDMAN\_2020\_S-G2 | Details ... | 273 | -0.49 | -3.00 | 0.000 | 0.000 | 0.000 | 1383 | tags=68%, list=26%, signal=87% |
| 19 | DOROSZKO\_U3054MG\_CLUSTER\_2 | Details ... | 274 | -0.48 | -2.99 | 0.000 | 0.000 | 0.000 | 606 | tags=39%, list=11%, signal=41% |
| 20 | RICHARDS\_VERHAAK\_CANCERCELL\_2010\_MESENCHYMAL | Details ... | 108 | -0.55 | -2.93 | 0.000 | 0.000 | 0.000 | 958 | tags=49%, list=18%, signal=59% |
| 21 | SUVA\_MES1\_PMID\_31327527 |  | 37 | -0.69 | -2.90 | 0.000 | 0.000 | 0.000 | 468 | tags=59%, list=9%, signal=65% |
| 22 | RICHARDS\_NEFTEL\_CELL\_2019\_MES1 |  | 37 | -0.69 | -2.89 | 0.000 | 0.000 | 0.000 | 468 | tags=59%, list=9%, signal=65% |
| 23 | GANDAL\_IBD\_UP\_PMID29439242 |  | 59 | -0.60 | -2.81 | 0.000 | 0.000 | 0.000 | 498 | tags=46%, list=9%, signal=50% |
| 24 | RICHARDS\_NOWAKOWSKI\_SCIENCE\_2017\_MGE\_DIV\_UPREG |  | 450 | -0.42 | -2.77 | 0.000 | 0.000 | 0.000 | 1537 | tags=60%, list=29%, signal=77% |
| 25 | FELDMAN\_2020\_LATEG1 |  | 74 | -0.56 | -2.76 | 0.000 | 0.000 | 0.000 | 721 | tags=61%, list=14%, signal=69% |
| 26 | GAROFANO\_IAVARONE\_PPR\_SUBTYPE |  | 294 | -0.43 | -2.68 | 0.000 | 0.000 | 0.000 | 1210 | tags=48%, list=23%, signal=59% |
| 27 | GANDAL\_AUTISM\_UP\_PMID29439242 |  | 67 | -0.55 | -2.64 | 0.000 | 0.000 | 0.000 | 887 | tags=55%, list=17%, signal=66% |
| 28 | VANGRONINGEN\_MES |  | 299 | -0.42 | -2.62 | 0.000 | 0.000 | 0.000 | 1071 | tags=44%, list=20%, signal=52% |
| 29 | SUVA\_G2M\_PMID\_31327527 |  | 41 | -0.60 | -2.61 | 0.000 | 0.000 | 0.000 | 1671 | tags=85%, list=32%, signal=124% |
| 30 | RICHARDS\_NEFTEL\_CELL\_2019\_G2\_M |  | 41 | -0.60 | -2.61 | 0.000 | 0.000 | 0.000 | 1671 | tags=85%, list=32%, signal=124% |
| 31 | DALMO\_BARCODENUMBER\_UNPUB |  | 155 | -0.46 | -2.60 | 0.000 | 0.000 | 0.000 | 1339 | tags=64%, list=25%, signal=83% |
| 32 | FELDMAN\_2020\_G2-M |  | 224 | -0.43 | -2.58 | 0.000 | 0.000 | 0.000 | 1289 | tags=62%, list=24%, signal=78% |
| 33 | NB\_KI\_CLUSTER11 |  | 43 | -0.58 | -2.51 | 0.000 | 0.000 | 0.000 | 880 | tags=56%, list=17%, signal=66% |
| 34 | RICHARDS\_NOWAKOWSKI\_SCIENCE\_2017\_MURAL\_UPREG |  | 433 | -0.38 | -2.46 | 0.000 | 0.000 | 0.000 | 815 | tags=32%, list=15%, signal=35% |
| 35 | LARSSON\_STATE\_1 |  | 75 | -0.49 | -2.45 | 0.000 | 0.000 | 0.000 | 910 | tags=53%, list=17%, signal=63% |
| 36 | SEGERMAN\_MULTITHERAPY\_RESISTANCE\_FIG5A\_PMID\_27974212 |  | 24 | -0.67 | -2.44 | 0.000 | 0.000 | 0.000 | 883 | tags=58%, list=17%, signal=70% |
| 37 | RICHARDS\_NOWAKOWSKI\_SCIENCE\_2017\_RG\_EARLY\_UPREG |  | 315 | -0.38 | -2.39 | 0.000 | 0.000 | 0.000 | 1476 | tags=53%, list=28%, signal=69% |
| 38 | RICHARDS\_NOWAKOWSKI\_SCIENCE\_2017\_MGE\_IPC1\_UPREG |  | 411 | -0.35 | -2.28 | 0.000 | 0.000 | 0.000 | 1551 | tags=51%, list=29%, signal=67% |
| 39 | FELDMAN\_2020\_NEURAL G0 |  | 303 | -0.36 | -2.28 | 0.000 | 0.000 | 0.000 | 914 | tags=44%, list=17%, signal=50% |
| 40 | RICHARDS\_NOWAKOWSKI\_SCIENCE\_2017\_MGE\_IPC2\_UPREG |  | 386 | -0.35 | -2.26 | 0.000 | 0.000 | 0.001 | 1296 | tags=50%, list=24%, signal=61% |
| 41 | PARADA\_T1\_UP\_26461091 |  | 60 | -0.46 | -2.12 | 0.000 | 0.000 | 0.010 | 1049 | tags=57%, list=20%, signal=70% |
| 42 | RICHARDS\_LIDDELOW\_NATURE\_2017\_A2\_REACTIVE\_ASTROCYTES |  | 28 | -0.53 | -2.11 | 0.000 | 0.000 | 0.014 | 591 | tags=54%, list=11%, signal=60% |
| 43 | RICHARDS\_NOWAKOWSKI\_SCIENCE\_2017\_IPC\_DIV1\_UPREG |  | 486 | -0.32 | -2.09 | 0.000 | 0.000 | 0.021 | 1252 | tags=42%, list=24%, signal=50% |
| 44 | SUVA\_MES2\_PMID\_31327527 |  | 32 | -0.48 | -2.00 | 0.000 | 0.001 | 0.053 | 1937 | tags=81%, list=37%, signal=127% |
| 45 | RICHARDS\_NEFTEL\_CELL\_2019\_MES2 |  | 32 | -0.48 | -1.96 | 0.000 | 0.001 | 0.077 | 1937 | tags=81%, list=37%, signal=127% |
| 46 | SINGH\_SOX2\_DOWNREGULATED\_TRANSCRIPTS\_PMID\_28122245 |  | 84 | -0.38 | -1.95 | 0.000 | 0.002 | 0.084 | 885 | tags=33%, list=17%, signal=39% |
| 47 | IVY\_CTPAN |  | 93 | -0.37 | -1.93 | 0.000 | 0.002 | 0.098 | 1037 | tags=41%, list=20%, signal=50% |
| 48 | RICHARDS\_NOWAKOWSKI\_SCIENCE\_2017\_CHOROID\_UPREG |  | 317 | -0.30 | -1.91 | 0.000 | 0.002 | 0.119 | 1319 | tags=38%, list=25%, signal=48% |
| 49 | RICHARDS\_NOWAKOWSKI\_SCIENCE\_2017\_MGE\_RG2\_UPREG |  | 304 | -0.30 | -1.90 | 0.000 | 0.002 | 0.135 | 1121 | tags=40%, list=21%, signal=48% |
| 50 | NB\_KI\_CLUSTER12 |  | 40 | -0.44 | -1.88 | 0.002 | 0.003 | 0.157 | 595 | tags=33%, list=11%, signal=36% |
| 51 | ALMSTEDT\_VGR\_DIFF |  | 483 | -0.28 | -1.85 | 0.000 | 0.003 | 0.199 | 770 | tags=28%, list=15%, signal=30% |
| 52 | RICHARDS\_CAHOY\_JNEUROSCI\_2010\_ASTRO\_YOUNG |  | 493 | -0.28 | -1.83 | 0.000 | 0.004 | 0.220 | 1094 | tags=37%, list=21%, signal=42% |
| 53 | NB\_KI\_CLUSTER16 |  | 80 | -0.36 | -1.82 | 0.002 | 0.004 | 0.228 | 806 | tags=35%, list=15%, signal=41% |
| 54 | DOROSZKO\_U3180MG\_CLUSTER\_4 |  | 101 | -0.33 | -1.75 | 0.002 | 0.007 | 0.372 | 1947 | tags=62%, list=37%, signal=97% |
| 55 | NB\_KI\_CLUSTER17 |  | 106 | -0.32 | -1.70 | 0.005 | 0.010 | 0.501 | 1009 | tags=39%, list=19%, signal=47% |
| 56 | RICHARDS\_ZHONG\_NATURE\_2018\_MICROGLIA\_UPREG |  | 160 | -0.30 | -1.69 | 0.003 | 0.011 | 0.523 | 593 | tags=23%, list=11%, signal=25% |
| 57 | KLING\_GBM\_PHENO\_PMID\_27667176 |  | 109 | -0.32 | -1.68 | 0.002 | 0.011 | 0.545 | 534 | tags=25%, list=10%, signal=27% |
| 58 | NB\_KI\_CLUSTER13 |  | 95 | -0.32 | -1.67 | 0.009 | 0.012 | 0.562 | 994 | tags=36%, list=19%, signal=43% |
| 59 | FELDMAN\_2020\_M-EARLYG1 |  | 83 | -0.33 | -1.65 | 0.009 | 0.014 | 0.629 | 794 | tags=43%, list=15%, signal=50% |
| 60 | DOROSZKO\_U3054MG\_CLUSTER\_6 |  | 69 | -0.33 | -1.64 | 0.007 | 0.015 | 0.661 | 1776 | tags=58%, list=34%, signal=86% |
| 61 | NB\_KI\_CLUSTER3 |  | 87 | -0.31 | -1.60 | 0.010 | 0.020 | 0.789 | 1660 | tags=47%, list=31%, signal=68% |
| 62 | ROSEN\_KRONA\_TI\_UNPUBL |  | 47 | -0.36 | -1.58 | 0.011 | 0.023 | 0.823 | 514 | tags=21%, list=10%, signal=23% |
| 63 | KRONA\_ROSEN\_WHITE\_MATTER\_INVASION |  | 160 | -0.28 | -1.56 | 0.002 | 0.026 | 0.870 | 498 | tags=19%, list=9%, signal=21% |
| 64 | ALMSTEDT\_17Q\_MSIGDB |  | 182 | -0.27 | -1.54 | 0.003 | 0.029 | 0.903 | 501 | tags=16%, list=9%, signal=18% |
| 65 | RICHARDS\_NOWAKOWSKI\_SCIENCE\_2017\_MICROGLIA\_UPREG |  | 421 | -0.24 | -1.54 | 0.000 | 0.030 | 0.914 | 996 | tags=28%, list=19%, signal=32% |
| 66 | DOROSZKO\_U3013MG\_CLUSTER\_1 |  | 32 | -0.37 | -1.51 | 0.053 | 0.034 | 0.940 | 144 | tags=19%, list=3%, signal=19% |
| 67 | DOROSZKO\_U3180MG\_CLUSTER\_6 |  | 29 | -0.39 | -1.51 | 0.061 | 0.034 | 0.941 | 967 | tags=52%, list=18%, signal=63% |
| 68 | ANGIOGENESIS\_GO\_0001525 |  | 17 | -0.43 | -1.46 | 0.092 | 0.047 | 0.984 | 252 | tags=29%, list=5%, signal=31% |
| 69 | KRONA\_ROSEN\_PERIVASCULAR\_INVASION |  | 47 | -0.31 | -1.38 | 0.083 | 0.076 | 0.999 | 452 | tags=21%, list=9%, signal=23% |
| 70 | GANDAL\_BIPOLAR\_UP\_PMID29439242 |  | 57 | -0.29 | -1.35 | 0.096 | 0.089 | 1.000 | 415 | tags=19%, list=8%, signal=21% |
| 71 | NB\_KI\_CLUSTER20 |  | 23 | -0.36 | -1.34 | 0.127 | 0.095 | 1.000 | 685 | tags=35%, list=13%, signal=40% |
| 72 | PARADA\_T2\_UP\_26461091 |  | 61 | -0.26 | -1.25 | 0.152 | 0.160 | 1.000 | 722 | tags=33%, list=14%, signal=38% |
| 73 | NB\_KI\_CLUSTER1 |  | 16 | -0.37 | -1.24 | 0.225 | 0.163 | 1.000 | 3054 | tags=94%, list=58%, signal=221% |
| 74 | ALMSTEDT\_11Q\_MSIGDB |  | 175 | -0.22 | -1.24 | 0.113 | 0.165 | 1.000 | 652 | tags=19%, list=12%, signal=21% |
| 75 | NB\_KI\_CLUSTER23 |  | 167 | -0.20 | -1.18 | 0.182 | 0.224 | 1.000 | 760 | tags=23%, list=14%, signal=26% |
| 76 | GANDAL\_IBD\_DN\_PMID29439242 |  | 21 | -0.32 | -1.16 | 0.267 | 0.242 | 1.000 | 494 | tags=29%, list=9%, signal=31% |
| 77 | GANDAL\_ALCOHOLADDICTION\_UP\_PMID29439242 |  | 81 | -0.19 | -0.98 | 0.485 | 0.552 | 1.000 | 800 | tags=21%, list=15%, signal=24% |
| 78 | NB\_KI\_CLUSTER14 |  | 159 | -0.16 | -0.92 | 0.623 | 0.659 | 1.000 | 1017 | tags=23%, list=19%, signal=28% |
| 79 | RICHARDS\_NOWAKOWSKI\_SCIENCE\_2017\_U3\_UPREG |  | 423 | -0.14 | -0.91 | 0.719 | 0.672 | 1.000 | 1315 | tags=27%, list=25%, signal=33% |
| 80 | RICHARDS\_VERHAAK\_CANCERCELL\_2010\_NEURAL |  | 36 | -0.21 | -0.89 | 0.600 | 0.712 | 1.000 | 89 | tags=6%, list=2%, signal=6% |
| 81 | DOROSZKO\_U3054MG\_CLUSTER\_9 |  | 257 | -0.12 | -0.70 | 0.977 | 0.953 | 1.000 | 404 | tags=7%, list=8%, signal=8% |
| 82 | RICHARDS\_NOWAKOWSKI\_SCIENCE\_2017\_GLYC\_UPREG |  | 417 | -0.09 | -0.59 | 1.000 | 0.998 | 1.000 | 1333 | tags=25%, list=25%, signal=31% |
| 83 | NB\_KI\_CLUSTER15 |  | 99 | -0.11 | -0.57 | 0.997 | 0.991 | 1.000 | 1852 | tags=39%, list=35%, signal=59% |
| 84 | NB\_KI\_CLUSTER24 |  | 65 | -0.10 | -0.49 | 0.998 | 0.993 | 1.000 | 2989 | tags=71%, list=56%, signal=161% |
Table: Gene sets enriched in phenotype **na **[plain text format]****
